# Effects of Different Beddings on Heifer Behaviors and Location Distributions in Lying and Elimination

**DOI:** 10.3390/ani15071009

**Published:** 2025-03-31

**Authors:** Bin Wu, Liyu Zhang, Guowen Li, Chongchong Zhao, Weiguang Hao, Peishi Yan, Xingming Yang, Shengjuan Wei

**Affiliations:** 1 College of Animal Science and Technology, Nanjing Agricultural University, Nanjing 210095, China; 2 Department of Green Development of Animal Husbandry Industry, National Animal Husbandry Station, Beijing 100125, China; 3 College of Resources & Environmental Sciences, Nanjing Agricultural University, Nanjing 210095, China

**Keywords:** behavior, lying, elimination, heifers, bedding

## Abstract

Different beddings result in alterations in barn environments and are responsible for changes in heifer behaviors and welfare. In this experiment, twenty-four Holstein heifers were randomly divided into four pens with various beddings, including sawdust (SD), fermented manure and sawdust (FSD), fermented manure and straw (FST), and a fermented manure mixture (FMM) for 31 days. The water content and surface layer temperature of the beddings were measured. We studied the activity budget, diurnal rhythms, and locations in lying and elimination of these heifers by videoing them for three consecutive days in the early and late stages of the experiment. Compared with the three fermented beddings (FSD, FST, and FMM), the SD bedding had a higher surface layer temperature and lower water content (*p* < 0.05). The SD bedding resulted in significantly less time ruminating (*p* < 0.01) and more time walking (*p* < 0.01) for heifers. The heifers in the SD and FMM bedding spent less time lying (*p* < 0.01) and more time standing (*p* < 0.01). Heifers in SD and FSD beddings presented lower scores in surface cleanliness rating (*p* < 0.01). Heifer activities, including eating, drinking, elimination, and lying, showed clear circadian patterns in four groups. Elimination behavior had a significant positive correlation with drinking/eating (*p* < 0.01). Moreover, similar location distributions in lying and elimination of heifers in various bedding pens were observed. Heifers preferred to eliminate the near eating area and drinking area and keep lying out of the eating region (*p* < 0.01). These findings could provide a reference for the selection of bedding materials and bedding management in dairy farming.

## Simple Summary

The rising costs of conventional bedding materials have made recycled manure solids increasingly favored as bedding alternatives. Different beddings might affect the behavior and welfare of heifers. This study aimed to evaluate heifer behaviors, cleanliness, and location distributions in lying and elimination on different beddings (SD, sawdust; FSD, fermented manure and sawdust; FST, fermented manure and straw; FMM, fermented manure mixture). Our results indicated that FSD bedding has the best beneficial effect in increasing the lying and ruminating time of heifers and improving surface cleanliness. Under different beddings, heifers showed similar circadian rhythms in eating, drinking, elimination, and lying behaviors, and elimination behavior was positively correlated with drinking/eating. Moreover, heifers preferred to lie away from eating regions and eliminate near eating and drinking locations. These findings provide a reference for the selection of bedding materials and bedding management in dairy farming, which might be the key factors to optimize cattle welfare.

## 1. Introduction

The housing system of dairy cows has changed significantly in contemporary dairy production. A modern approach, known as the compost-bedded pack (CBP) barns, which integrates the resting, exercising, and excreting areas on the microbial fermentation bedding, has become popular [1]. The CBP possesses the advantages of providing more comfortable space and better welfare for cows, as well as reducing the costs of cattle shed construction and slurry treatment, gradually replacing the conventional tie-stall or free-stall cubicle housing [2]. The first CBP system was developed by dairy producers in Virginia during the 1980s in the United States. Internationally, countries such as Israel, Germany, Austria, etc., have also begun adopting CBP systems as viable housing options for dairy cattle [1].

In the CBP system, the bedding is often constructed using agricultural and forestry by-products, such as straw, wood chips, rice husks, etc. However, with the increased demand for organic wastes in the energy market and the rising transportation costs, the prices of traditional bedding materials are rising, restricting the application of the CBP housing system [3]. An increasing number of farms are using recycled manure solids (RMS) as alternative bedding materials for dairy cows to better control the availability and cost of bedding materials [4,5]. The RMS could be used as bedding materials in both lying stalls and CBP bedding. However, it must be noted that in the absence of proper pre-treatment, residual pathogenic microorganisms can pose a significant risk to the health of cows. At present, the RMS technology, which began in the 1970s, has been widely adopted in several countries, including the United States, New Zealand, the United Kingdom, and Canada [3].

Bedding material is an important part of indoor housing systems for livestock, exerting a profound influence on animal health and behavior. Different bedding materials and farming conditions can significantly influence the behavior and well-being of dairy cows. Dairy cows are highly motivated to lie down and eat [6,7]; longer lying or rumination time is often considered an indicator of better welfare [8,9]. Bedding material plays an essential role in enhancing cattle comfort and modulating their lying behavior. For instance, cows in cubicles have shown a preference for RMS bedding over straw, sawdust, and sand [10]. In the CBP system, compared with chaff bedding, fermented manure and chaff bedding have been shown to remarkably improve the lying behavior and hygiene of buffaloes while simultaneously reducing bedding costs [11].

The daily rest and activity patterns of cows serve as potential markers of individual animal health status [12] and reflect how well farm animals have adapted to their surroundings [13]. Monitoring these behavior patterns can be used to improve production management and maximize on-farm efficiency and animal welfare in feedlot settings [14]. In addition, daily and seasonal variations, modulated by the day-night cycle, prompt organisms to adapt, which is manifested as rhythmic biological and behavioral patterns [15]. Dairy cows tend to exhibit better general health and productivity when their environments and feeding schedules align with their natural cycles [16]. Therefore, it is critical to comprehend animal rhythms to optimize management and ensure their well-being.

Cows exhibit different behavioral regions in various farm systems. Existing research on the behavioral regions of cows has been mainly conducted in pastures, with little research in indoor housing systems. Tie-stall and free-stall cubicle housings are designed to separate the lying animal from its excreta by movement limitation or functional partitioning [17]. In contrast, in the CBP system, such restrictions are absent. For cows, tie-stalls have a greater percentage of contaminated stalls compared with cubicles [18]. When comparing the two housing systems of straw yards and cubicle systems, different excretion rates appear in the lying area [19]. Cows generally prefer soft and dry bedding, as it offers a more comfortable lying surface [20]. However, lying preferences can change due to environmental conditions [21]. Confirmation of cow behavioral preferences could be tested by observing the regional distribution of cow behavior [22]. Location data concerning lying and elimination can provide animal activity information and have many applications in improving animal welfare.

Currently, research on the effects of diverse bedding materials on the circadian rhythms and detailed spatial distribution of lying and elimination behaviors of heifers within the CBP system remains scarce. This study aims to fill this research gap by comparing the application effects of sawdust and three types of RMS (produced through aerobic fermentation of manure and agricultural and forestry by-products) on the behavior and welfare of heifers in the CBP system. The findings are expected to optimize farming management and improve the living conditions of heifers, thereby enhancing animal welfare.

## 2. Materials and Methods

### 2.1. Animals and Experimental Pens

**Figure 1:**
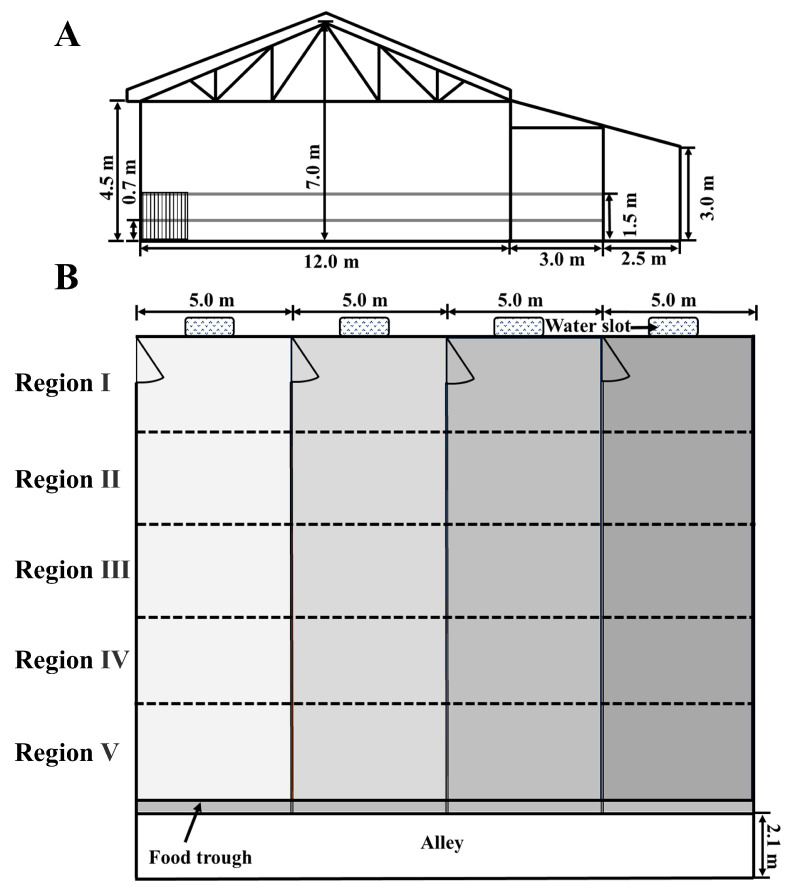
Schematic view of the experimental pens. (**A**) the side view of the barn. (**B**) the top view of the barn. The areas with colors ranging from light to dark represent SD, FMM, FSD, and FST, respectively. SD = sawdust; FMM = fermented manure mixture; FSD = fermented manure and sawdust; FST = fermented manure and straw.

This experiment was performed with permission from the Committee of the Animal Research Institute of Nanjing Agricultural University, China (SYXK2011-0036). The study was performed from 28 August to 27 September 2022 in a dairy farm in Zhenjiang City, Jiangsu Province, China. Twenty-four healthy Holstein heifers (6–7 months of age) were used in the experiment, with the initial body weight of 223.86 ± 3.68 kg. The temperature was 14–34 °C throughout the experiment, with an average temperature of 24 °C, the relative humidity 54.94–88.04%, and the wind speed 0.63–1.66 m/s. The heifers were weighed and then divided into four different bedding treatment groups, with 6 heifers in each group balanced by body weight and age. The barn was oriented east-west, facing south, and is a double-slope front-and-back-open structure (Figure 1). Four pens in the single-row house were situated side by side, with a feeding alley outward. Pens were separated by a metal fence that allowed contact among animals of adjoining pens. The dimension for each pen was 15 × 5 m. A water trough was mounted on a 0.7 m high concrete slab at the north side of each pen. All heifers had the same management regimen. Heifers were fed twice each day by one keeper at 07:40 and 14:40. All heifers were fed total mixed ration (TMR) ad libitum and had free access to water. The nutrients (DM basis) of the feed contained 15.13% CP, 1.69% EE, 45.6% NDF, 30.0% ADF, 5.57% ash, 0.73% Ca, and 0.73% P.

### 2.2. Bedding Material Preparation and Management

Bedding materials were made from cow manure collected on the farm as well as sawdust and wheat straw near the farm. For the fermented manure and sawdust (FSD), the ratio of sawdust to manure was 3:1; for the fermented manure and straw (FST), the ratio of straw to manure was 3:1; and for the fermented manure mixture (FMM), the ratio of sawdust, straw, and manure was 2:1:1. Materials were mixed with a forklift to adjust the moisture content to about 65% in a pile. The characteristics of the mixed materials are as follows: FSD, moisture 68.07%, organic matter (OM) 76.11%, total nitrogen (TN) 1.01%, and C/N 43.87; FST, moisture 67.23%, OM 71.91%, TN 1.16%, and C/N 36.03; FMM, moisture 67.38%, OM 67.25%, TN 1.05%, and C/N 37.10. The sawdust (SD) had a moisture content of 13.94%, OM of 79.43%, TN of 0.26%, and C/N of 176.52. After the fermentation temperature first rose above 50 °C, the piles were turned by a forklift every three days to supply oxygen. Fermentation was terminated when the pile temperature exceeded 50 °C for 7 days and the water content dropped to about 50%. Then, the piles were spread out to dry until the water content declined to about 30%. The SD, FSD, FST, and FMM were then transferred into the corresponding four pens (25 cm in thickness). Separators were used between compartments to prevent substrate mixing. During the feeding period, the bedding was tilled every five days with a rototiller.

### 2.3. Determination of Surface Temperature and Water Content of the Bedding

The surface layer temperature at a depth of 1.5 cm was monitored using a thermocouple thermometer (YC-747UD, Yuqing Technology Co., Ltd., New Taipei City, Taiwan, China). Measurements were taken at 09:00 and 15:00 every 5 days, following a five-point sampling method. The values obtained within each pen were averaged. Bedding samples with full depth were collected every 5 days at the same position where the temperature was measured, followed by thorough mixing. The water content of bedding materials was determined via the drying method. Weight loss after drying to constant weight at 105 °C was recorded to calculate the water content.

### 2.4. Behavioral Observation

The heifer’s behavior was video-recorded continuously in a real-time mode by a network video recorder (DS-7808N-F1, Hikvision, Hangzhou Hikvision Digital Technology Co., Ltd., Hangzhou, China) with eight cameras (DS-IPC-B12HV3-IA, Hikvision) for 6 days (two continuous 72 h periods). Video footage was sampled on 3–5th September (the early period) and 24–26th September (the late period). During the chosen days, human interventions such as bed management and data collection inside the pen were avoided. The chosen 6 days were cloudy, and the outside temperature was 17–30 °C, with an average temperature of 23 °C. Each pen had two cameras, with one on the north side and the other on the south side to avoid any visual blind spot. Cameras were set 3.0 m high above the ground. Individual behavioral data were collected from the video by using the scan-sampling method. Heifers were observed individually via the focal-animal sampling, and then the behavioral observation was performed by the same person using the continuous focus observation method. A total of 8 behaviors of heifers were recorded in the experiment. The definition of each behavior is described in Table 1 [4,15]. Each pen was composed of 5 regions to observe, and the size of one region was 15 m^2^ (Figure 1). Regions were recorded where the lying and elimination behaviors occurred, respectively. Animals on the border were counted in the region where the majority of their bodies were located. Night-time was defined as the period between sunset and sunrise [23].

### 2.5. Body Surface Hygiene

The body surface hygiene of heifers was observed manually and scored. During the morning feeding period of the last three days of the experiment, individuals were observed and analyzed by evaluating the cleanliness of the udder and lower and upper hind legs. According to the hygiene scoring system [24], heifers were scored by a 4-point cleanliness scale with 1 = very clean to 4 = very dirty. Specifically, 1 = 0%; 2 = 0–10%; 3 = 10–30%; and 4 = more than 30% of the body surface contaminated with manure (including splashes of feces and caked-on dirt).

### 2.6. Statistical Analysis

Statistical analysis was carried out using the IBM SPSS statistics (version 26.0, IBM Corporation, New York, NY, USA). To check the data normality, the Kolmogorov–Smirnov test was used. The *p*-value greater than 0.05 was considered the data conformed to a normal distribution. All data were expressed as the mean  ±  standard error of the mean (SEM). Behavioral data may be affected by individual differences, temporal sequence effects, or group effects. The total time of one day (24 h) for each behavior was analyzed with a completely random design with repeated measurements using the MIXED procedure, which could handle within-subject variability effectively. Data were adjusted with the data of the period as a covariate factor. The model included the fixed effects of bedding materials (SD, FSD, FST, and FMM), time (early-phase and late-phase), bedding materials × time, and covariates. For the diurnal rhythm analysis, the behavior duration and the elimination number per hour were calculated, respectively. During the analysis of circadian rhythms of behavior, the repeated general linear model (GLM) test was employed to identify significant differences in the behavior duration and the event number in each hour among the 24 h with an hour effect as the within-subject factor. The F-value was used to test whether there were significant differences in the duration of behavior and the frequency of elimination among different treatment groups for each hour within the 24 h period. Heifers were regarded as independent units for the statistical analysis. The correlation between eating and elimination, as well as elimination and drinking, was analyzed using the Pearson product-moment correlation coefficient. One-way ANOVA analysis was used for environmental indicators and cleanliness assay. The Tukey HSD test was employed to evaluate the differences between any two groups. *p*-values less than 0.05 and 0.01 were considered significantly different and very significantly different, respectively.

## 3. Results

### 3.1. Environmental Indicators of Four Bedding Groups

**Figure 2:**
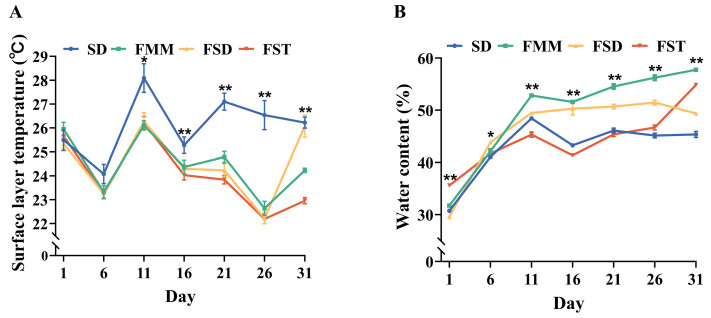
The microenvironment of four bedding groups. (**A**) surface layer temperature and (**B**) water content of beddings. * *p* ˂ 0.05 and ** *p* ˂ 0.01. SD = sawdust; FMM = fermented manure mixture; FSD = fermented manure and sawdust; FST = fermented manure and straw.

The surface layer temperature of the beddings ranged from 22.19 to 28.09 °C throughout the experimental period (Figure 2A). During the first day, the surface layer temperatures of all four bedding materials were between 25 and 26 °C (*p* > 0.05). Compared with the three fermented bedding groups, the SD presented a higher surface layer temperature during d 11–31 (*p* < 0.05 or *p* < 0.01). The water content was maintained at 29.55–57.74% throughout the test period, with an increasing trend in the four bedding groups (Figure 2B). The SD and FST had lower water content during d 11–26 than FSD and FMM (*p* < 0.01), and SD possessed the lowest water content at d 31 among the four groups.

### 3.2. Effect of Bedding Type and Time on Heifer Behaviors

The behaviors of heifers are listed in Table 2. The types of bedding materials had different effects on drinking, eating, ruminating, standing, lying, walking, and fighting (*p* < 0.05 or *p* < 0.01), with no effect on the number of eliminations (*p* > 0.05). Compared to SD, the three fermented beddings promoted ruminating (*p* < 0.01) and reduced walking (*p* < 0.01) behaviors of heifers. The FST and FSD increased the lying time of heifers (*p* < 0.01), with lower standing time observed (*p* < 0.01). Compared to the FST, the FMM heifers increased the drinking time (*p* < 0.01). The eating time of the FSD was lower than the FST (*p* < 0.05). The fight time of the FSD was higher than the FST (*p* < 0.05). The time had different effects on ruminating, standing, lying, and walking (*p* < 0.01), with no effects on elimination, drinking, eating, and fighting (*p* > 0.05). There was an interaction between time and bedding type in standing and lying (*p* < 0.01).

### 3.3. Cleanliness of Heifers

The body surface cleanliness is shown in Table 3. Results showed the cleanliness score of both FMM and FST groups was significantly higher than that of SD and FSD groups (*p* < 0.01). No difference was observed between SD and FSD (*p* > 0.05).

### 3.4. The Circadian Rhythm of Behaviors

**Figure 3:**
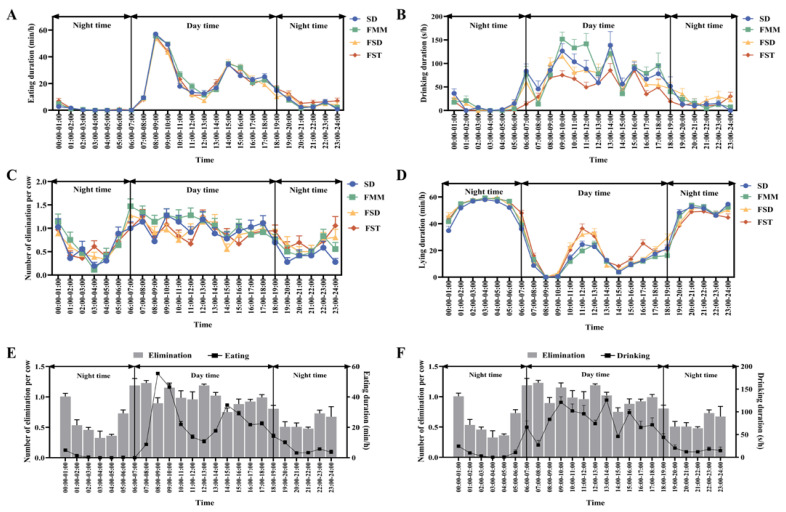
The circadian rhythm of heifer behaviors. Daytime is set from 06:00 to 19:00; the rest of the time is nighttime. (**A**) eating duration, (**B**) drinking duration, (**C**) numbers of elimination, (**D**) lying duration, (**E**) correlations of elimination and eating, and (**F**) correlations of elimination and drinking. SD = sawdust; FMM = fermented manure mixture; FSD = fermented manure and sawdust; FST = fermented manure and straw.

Figure 3 showed the heifers circadian rhythm of eating, drinking, elimination, and lying behaviors of four bedding groups within 24 h, showing a spiral structure with up and down fluctuations. Marked circadian variations in the eating behavior were shown in Figure 3A (SD, F = 93.44; FMM, F = 100.88; FSD, F = 85.76; FST, F = 62.10; *p* < 0.01). Two peaks of the eating time appeared after 07:00 and 14:00. The percentage of 90.02 ± 1.40% of the eating behavior took place in the daytime, and the remaining 9.98 ± 1.40% occurred in the night (*p* < 0.01). Marked circadian variations in the drinking behavior were shown in Figure 3B (SD, F = 14.12; FMM, F = 13.92; FSD, F = 11.42; and FST, F = 13.06; *p* < 0.01). Drinking time in FMM and FSD groups peaked at 09:00–10:00, with SD and FST groups peaking at 13:00–14:00. The percentage of 88.41 ± 1.50% of the drinking behavior took place in the daytime, and the remaining 11.59 ± 1.50% occurred in the night (*p* < 0.01). Marked circadian variations in the elimination behavior were shown in Figure 3C (SD, F = 6.49; FMM, F = 6.53; FSD, F = 3.76; and FST, F = 3.83; *p* < 0.01). More than 2777 eliminative behavior events (SD, 653 times; FMM, 743 times; FSD, 688 times; FST, 693 times) were observed. The elimination frequency was 1.23 ± 0.07 n/h. Elimination events exhibited a daily periodical pattern, and the peak occurred from 06:00 to 10:00, with 67.27 ± 1.71% of the elimination behavior in the daytime and the remaining 32.73 ± 1.71% in the night (*p* < 0.01). Marked circadian variations in the lying behavior were shown in Figure 3D (SD, F = 114.10; FMM, F = 113.29; FSD, F = 94.30; FST, F = 80.95; *p* < 0.01). The peak lying behavior of heifers occurred at 03:00–05:00 in the nighttime, with a peak at 11:00–13:00 in the daytime. The proportion of lying in the daytime was significantly lower than that in the night (27.02 ± 1.81% in the daytime and 72.98 ± 1.81% in the night; *p* < 0.01). Furthermore, there were positive correlations between elimination and eating (R2 = 0.15, *p* < 0.01; Figure 3E) as well as between elimination and drinking (R2 = 0.20, *p* < 0.01; Figure 3F).

### 3.5. The Location Distribution of Lying and Elimination

**Figure 4:**
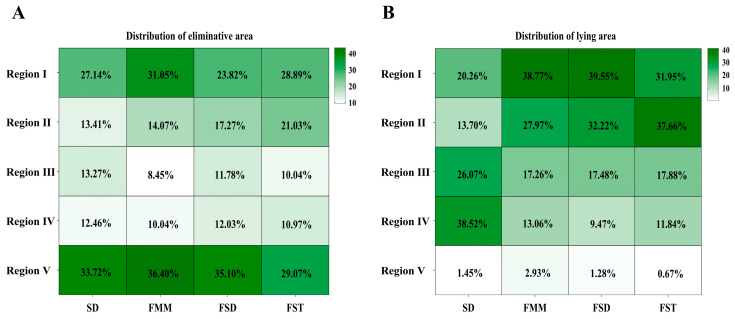
Distribution of eliminative and lying regions. Each pen was equally divided into five regions, namely regions I, II, III, IV, and V, from the water slot side to the food trough side. (**A**) distribution of eliminative area; (**B**) distribution of lying area. SD = sawdust; FMM = fermented manure mixture; FSD = fermented manure and sawdust; FST = fermented manure and straw.

Distribution of heifers’ lying and elimination areas showed an obvious regional division in the CBP system (Figure 4). A similar pattern was observed in elimination area distributions of four groups. More than 50% of the heifer elimination occurred near the water slot and food trough (Figure 4A). Specifically, regions I and V had higher elimination frequency than other regions (*p* < 0.01), and the elimination frequencies of regions I and V accounted for 27.14% and 33.72% in the SD, 31.05% and 36.40% in the FMM, 23.82% and 35.10% in the FSD, and 28.89% and 29.07% in the FST, respectively. In the SD group, regions III and IV accounted for 64.59% of the total lying time, while the FMM, FSD, and FST groups spent 66.74%, 71.77%, and 69.61%, respectively, of the entire lying time at regions I and II (Figure 4B). All four groups spent less than 3% of the whole lying time in region V (*p* < 0.01).

## 4. Discussion

Dairy cow welfare refers to the production and living of dairy cows in a suitable environment, thereby reducing their risk of disease and achieving the best state of lactation and reproductive performance. Cows need to spend at least 50–60% of their time lying down and resting every day [25]. Given that cows usually ruminate while lying down, a reduction in lying time implies a corresponding decrease in rumination time [26]. The lying and ruminating time of cows are direct reflections of the comfort level of the bedding. Various factors, such as bedding temperature, surface texture, microbial activity, softness, and humidity, may all play a role in the comfort level of the bedding, thus impacting the lying and ruminating behaviors of heifers. Bedding temperature is a key environmental indicator affecting the comfort level of animals [27].

It has been reported that in a high-temperature environment, cows will increase their daily standing time and reduce their lying time [28,29] as well as their ruminating time [30]. In this study, the surface layer temperature of the SD bedding was significantly higher than that of the fermented bedding groups, and the significantly lower ruminating time of heifers in the SD group was observed. It is possible that the sawdust bedding contains a large amount of organic matter, which is easily decomposed by microorganisms, releasing a large amount of heat [2] and thus increasing the temperature of the bedding. The thermoneutral zone for dairy cows is generally considered to range from 5 to 25 °C [27]. As the surface temperature of the bedding in the SD group was above 25 °C during d 11–31, it might cause discomfort to heifers due to temperature issues. However, the underlying mechanisms for the change in heifer behavior need to be further explored. In CBP application, bedding fermentation heat exacerbating animal heat stress is a common concern during summer. Therefore, we compared the effects of different bedding materials on heifer behaviors during high temperatures. However, since bedding with high humidity and low temperature in winter can also pose problems for animals, conducting trials in other seasons will provide more comprehensive data for bedding selection. In addition, the location in the barn might affect the surface temperature of the bedding, such as solar radiation and wind direction. In order to mitigate the climatic differences caused by the positions of different groups, setting up replicate experiments and alternately placing the bedding in compartments can enhance the reliability of the experiment.

The softness and thickness of bedding also affect its comfort level. After fermentation, the bedding becomes softer, providing a more comfortable lying environment for cows [3], which may be another factor contributing to the increase in the lying and rumination time of cows. A previous study shows that compared with rice husk bedding, fermented manure bedding significantly increases the lying behavior of buffaloes [11]. In this experiment, heifers using FSD and FST fermented bedding spent more time lying and ruminating, indicating that the FSD and FST bedding are more comfortable compared to the SD and the FMM. Since different bedding thicknesses can also affect the lying and rumination time of cattle [4], in subsequent research, the optimal thickness for using FSD and FST should be further explored.

The cleanliness of cattle is an indicator of animal welfare, as it provides information about animal life quality and farm facility conditions [31]. Studies have shown an association between cleanliness and diseases in cows [32]. Cows in dirtier stalls have a greater possibility of being lame [33]. Compared with rubber mats and mattress bedding, cows on sand show better cleanliness, fewer integumentary changes on the hocks (such as hairless patches, lesions, and swellings), and a lower incidence of lameness [32]. When the udders and hind legs of a dairy cow are dirty, the somatic cell count (SCC) will increase, thereby increasing the risk of mastitis [24,34]. In this study, the cleanliness of the SD and FSD groups was significantly better than that of the FST and FMM groups. This may be attributed to the fact that straw has a low capacity to absorb moisture, and heifers lying on straw bedding are more likely to be contaminated with manure [35]. This result is similar to the situation in the free-stall system, in which cows using RMS as bedding material exhibited cleaner udders and legs compared to those bedded with straw [36].

Different bedding materials possess unique properties. Only by implementing proper daily management can we maintain the stability of the bedding. Sawdust has excellent absorption capabilities and can effectively absorb the manure of dairy cows. Additionally, the large surface area provided by the sawdust supports extensive microbial growth, facilitating the decomposition of the manure added daily by the heifers [2]. However, sawdust has a high proportion of organic matter, which can be utilized by microorganisms to generate heat, potentially increasing the risk of heat stress in dairy cows in high-temperature environments. RMS, which is soft, non-abrasive, and readily available, contributes to increased comfort for cows, longer lying times, and fewer hock lesions [3]. However, if the pre-treatment process is not thorough, there is a risk of pathogen residue. Furthermore, treading and tilling actions can facilitate the mixing of fresh manure, promoting the microbial decomposition of organic matter in the bedding materials. These activities also enhance the evaporation of moisture within the bedding. The research suggested that the moisture content should be within the range of 40% to 60% before adding new bedding [1]. Regular cleaning and replacement of bedding materials are also important bedding management measures.

In order to adapt to alterations in the circadian environment, animals exhibit a variety of daytime and nighttime behavioral patterns. It has been demonstrated that environmental elements have an impact on the circadian rhythm [37]. The daily activities of cows in this experiment, including eliminating, eating, drinking, and lying down, showed clear circadian patterns. Animals allocate their time among daily activities so that the cost of adapting to the environment is minimized [38]. In this experiment, heifers spent the most time exhibiting lying behavior. Heifers in all groups had a similar pattern of lying, with more than 70% of lying behavior occurring at night. This observation is consistent with a previous report, which indicates a temporal cyclicity pattern in lying behavior [39]. Therefore, in the management of heifers, it is essential to minimize nocturnal disturbances to prevent adverse effects on the animals. Previous studies have shown that elimination behavior shows clear circadian trends [23]. The elimination of heifers in the experiment also demonstrated a day-night rhythm, with over 60% of the elimination occurring during daytime and the peak elimination occurring between 7:00 and 8:00. We detected a markedly positive correlation between elimination and drinking/eating. Eating behavior has been observed to follow a circadian rhythm [23]. Generally, cattle grazing takes place in daylight, with major periods around the sunrise and sunset [40]. Intense eating activity at sunset may help to hold a steady release of nutrients to keep satiety overnight [41]. In a housed situation where feed is plentiful, cows do not need to worry about competition for food, thus allocating the evening feeding peak to other time periods. In the present experiment, the feeding behavior mainly occurred during daylight, with the peak of eating appearing after two manual feedings. Therefore, it is of great assistance in optimizing feeding management and enhancing animal welfare by mastering the circadian rhythm activities of heifers.

The behavior of fecal avoidance or location preference may impact the area where an animal chooses to excrete. Commonly, animals avoid the deposited feces or urine after eliminating. Fecal avoidance is an effective method for grazing animals to lower the risk of ingesting parasites [42]. In our study, heifers in four groups chose to lie away from their most excretory areas. However, there is no absolute negative correlation between the lying position and the excretory position, indicating heifers develop a certain tolerance for excreta when choosing a lying area in limited indoor areas. In this study, heifers in fermented groups preferred to lie near the water slots, while heifers in the SD group lay away from the feeding and drinking area. This may be caused by factors such as the location of the pen and the bedding characteristics. Elimination behavior of dairy cows displayed different regional distributions in cubicle or straw yard housing systems [19]. Furthermore, we found that different beddings in the CBP system did not affect the elimination distribution. The rate of heifer elimination was significantly greater in regions near eating and drinking areas than in other regions, which is in accordance with a previous study in a straw yard system [43]. Therefore, a paved walking area in front of the feeding/drinking trough could reduce the frequency of bedding replacement and improve efficiency by regularly cleaning the dung with a scraper. However, the cost of the corresponding manure—removal facilities and the risk of hoof diseases due to fecal contact should also be taken into comprehensive consideration.

## 5. Conclusions

In conclusion, during the experimental period, the three fermented beddings had lower surface temperatures than SD bedding. The FSD bedding had the best beneficial effect in terms of increasing lying and rumination time as well as improving cleanliness, thus enhancing heifer welfare. Heifers in different bedding groups showed similar and obvious circadian patterns in eating, drinking, elimination, and lying. Elimination behavior was positively correlated with drinking/eating behavior. Under the CBP system, heifers lay in places away from eating regions and preferred to eliminate in eating and drinking zones. Their elimination sites were determined by the functional areas of the room layout, not the substrate. Suitable bedding materials and the CBP system equipped with a scraper passage to enhance the living conditions of heifers may be a fairly good approach. Conducting trials in other seasons and with replicate experiments is needed in further research.

## Figures and Tables

**Table 1:** Descriptive ethogram used to record behavioral time budget data for heifers.

Observed Variables	Definition
Elimination	Defecated or urinated
Drinking	Heifer lowers its head to drink water from the sink
Eating	Head down close to forages or feedstuff, taking bites or chewing without regurgitating
Ruminating	Regurgitating or chewing on bolus that has been regurgitated
Standing	Standing with all 4 feet on the ground, the hoof and legs support the body
Lying	Without support of any leg and with the belly in contact with the bedding
Walking	The leg slowly alternates to move the body forward without any expression of other behaviors
Fighting	Striking another individual with the head or shoulder
Other behaviors	All behaviors except for the above behaviors (e.g., mounting, communication, etc.).

**Table 2:** Behavior characteristics of heifers in different bedding material groups (within 24 h).

Items	Bedding Treatment					*p*-Value		
	SD	FMM	FSD	FST	SEM	B	T	B × T
Elimination, n	18.14	20.64	19.11	19.25	0.33	0.07	0.33	0.35
Drinking, min	20.04 ^ab^	21.97 ^a^	18.01 ^ab^	16.36 ^b^	0.52	<0.01	0.87	0.38
Eating, min	324.18 ^ab^	337.29 ^ab^	315.64 ^b^	339.69 ^a^	3.09	0.02	0.93	0.62
Ruminating, min	429.82 ^b^	466.17 ^a^	478.07 ^a^	478.59 ^a^	3.97	<0.01	<0.01	0.57
Standing, min	692.38 ^a^	684.72 ^a^	635.57 ^b^	631.44 ^b^	3.78	<0.01	<0.01	<0.01
Lying, min	747.68 ^b^	755.29 ^b^	804.43 ^a^	808.56 ^a^	3.78	<0.01	<0.01	<0.01
Walking, min	20.97 ^a^	11.17 ^b^	10.69 ^b^	11.22 ^b^	0.25	<0.01	<0.01	0.36
Fighting, min	7.37 ^ab^	7.18 ^ab^	8.43 ^a^	4.72 ^b^	0.42	0.02	0.50	0.39

^a, b^ Values with different lowercase letters in the same row mean significantly different (*p* < 0.05). Bedding materials: SD = sawdust; FMM = fermented manure mixture; FSD = fermented manure and sawdust; FST = fermented manure and straw. Treatment effects: B = effect of bedding treatment; T = effect of measurement time; B × T = interaction between bedding treatment and measurement time.

**Table 3:** Scoring of surface cleanliness of heifers.

Items	Bedding Treatment				SEM	*p*-Value
	SD	FMM	FSD	FST		
Upper	2.17 ± 0.41 ^b^ (2/3)	3.33 ± 0.82 ^a^ (2/4)	2.17 ± 0.75 ^b^ (1/3)	3.67 ± 0.52 ^a^ (3/4)	0.19	<0.01
Lower	2.17 ± 0.41 ^b^ (2/3)	3.50 ± 0.55 ^a^ (3/4)	2.33 ± 1.03 ^b^ (1/4)	3.83 ± 0.41 ^a^ (3/4)	0.19	<0.01
Udder	1.83 ± 0.75 ^b^ (1/3)	3.17 ± 0.98 ^a^ (2/4)	1.83 ± 0.41 ^b^ (1/2)	3.50 ± 0.55 ^a^ (3/4)	0.21	<0.01

The results are expressed as the mean ± standard deviation (min/max). ^a, b^ Values with different lowercase letters in the same row mean significantly different (*p* < 0.05). Bedding materials: SD = sawdust; FMM = fermented manure mixture; FSD = fermented manure and sawdust; FST = fermented manure and straw. Observation position: Upper = upper and lateral hind legs; Lower = lower hind legs; Udder = around the udder. 1 = very clean and 4 = very dirty.

## Data Availability

The data presented in this study are available upon request from the corresponding author.

## Abbreviations

RMS, recycled manure solids; CBP, compost-bedded pack; SD, sawdust; FSD, fermented manure and sawdust; FST, fermented manure and straw; FMM, fermented manure, sawdust and straw; TMR, total mixed ration; DM, dry matter; CP, crude protein; EE, ether extract; NDF, neutral detergent fiber; ADF, acid detergent fiber; OM, organic matter; TN, total nitrogen.
